# Identifying treatment non-responders based on pre-treatment gait characteristics - A machine learning approach

**DOI:** 10.1016/j.heliyon.2023.e21242

**Published:** 2023-10-23

**Authors:** Rosa M.S. Visscher, Julia Murer, Fatemeh Fahimi, Elke Viehweger, William R. Taylor, Reinald Brunner, Navrag B. Singh

**Affiliations:** a Laboratory for Movement Biomechanics, Institute for Biomechanics, Department of Health Science & Technology, ETH Zürich, Zürich, Switzerland; b Department of Biomedical Engineering, University of Basel, Basel, Switzerland; c Singapore-ETH Centre, Future Health Technologies Program, CREATE campus, 1 CREATE Way, #06-01 CREATE Tower, Singapore 138602; d Laboratory of Movement Analysis, University Children's Hospital Basel (UKBB), Basel, Switzerland

**Keywords:** Clinical gait analysis, Treatment outcomes, Machine learning, Cerebral palsy

## Abstract

**Background:**

Paediatric movement disorders such as cerebral palsy often negatively impact walking behaviour. Although clinical gait analysis is usually performed to guide therapy decisions, not all respond positively to their assigned treatment. Identifying these individuals based on their pre-treatment characteristics could guide clinicians towards more appropriate and personalized interventions. Using routinely collected pre-treatment gait and anthropometric features, we aimed to assess whether standard machine learning approaches can be effective in identifying patients at risk of negative treatment outcomes.

**Methods:**

Observational data of 119 patients with movement disorders were retrospectively extracted from a local clinical database, comprising sagittal joint angles and spatiotemporal parameters, derived from motion capture data pre- and post-treatment (physiotherapy, orthosis, botulin toxin injections, or surgery). Participants were labelled based on their change in gait profile score (GPS, non-responders with a decline in GPS of <1.6° vs. responders). Their pre-treatment features (sagittal joint angles, spatiotemporal parameters, anthropometrics) were used to train a support vector machine classifier with 5-fold cross-validation and Bayesian optimization within a MATLAB-based Classification Learner App.

**Results:**

An average accuracy of 88.2 ± 0.5 % was achieved for identifying participants whose gait will not respond to treatment, with 64 % true negative rate and an area under the curve of 88 %.

**Conclusion:**

Overall, a classical machine learning model was able to identify patients at risk of not responding to treatment, based on gait features and anthropometrics collected prior to treatment. The output of such a model could function as a warning signal, notifying clinicians that a certain individual might not respond well to the standard of care and that a more personalized intervention might be needed.

## 1 Introduction

With a prevalence rate of around 2.11 per 1000 live births, cerebral palsy (CP) is the most common motor disability in childhood in the western world [1]. While brain lesions that cause CP are non-progressive, orthopaedic manifestations get worse over time, often leading to limited mobility and participation, which negatively influences the quality of life (QoL) in those affected [2].

There are multiple treatment options to optimize QoL by improving walking ability. To assist clinicians in making treatment decisions for improving walking ability in CP, clinical gait analysis (CGA) was introduced more than two decades ago [3]. Since then, CGA has become a standard approach for assessing movement disorders in developed countries. CGA consists of three-dimensional motion capture (kinematics, movement trajectories) together with the collection of ground reaction forces (kinetics) and measurements of muscle activation patterns (electromyography, EMG). While CGA has provided added value to many clinical decision-making processes [4] and led to improved treatment outcomes [5], it has not resulted in the expected improvement in patient satisfaction [6]. In addition, some individuals still worsen their gait after treatment that aimed to improve their walking ability. A possible reason for such a discrepancy, might be due to the high dimensional data, which makes the resulting kinematics and kinetics difficult to interpret and might therefore limit its impact on clinical decision making and setting of expectations.

Currently, mainly kinematic features and overall summary scores for gait and motor development are extracted from CGA to assist in treatment recommendations. However, it is unclear whether these parameters capture the full complexity of gait and motor development [7]. Advanced approaches for evaluating CGA outcomes through machine learning provide suitable alternatives to summarize this complexity and estimate treatment outcomes [8,9]. Moreover, such approaches could plausibly identify individuals at risk of showing no response under standard of care. To this end, previous research has already shown promising results [[10], [11], [12], [13], [14], [15], [16]], however most of these approaches are only applicable to specific interventions or cohorts, limiting their applicability in clinical settings. Additionally, there is a need for making model outputs more explainable and easier to interpret so that the approaches can be translated into clinical practice where they can assist clinicians in their decision-making processes.

Therefore, our study aims at investigating whether a standard machine learning approach would be able to identify patients at risk of showing no response in their gait after receiving standard treatment in Switzerland based on pre-treatment characteristics. Our primary objective was to evaluate model performance. The secondary objective was to highlight which input parameters were most relevant for the model's recommendation.

## 2 Materials and methods

### 2.1 Participants

For this observational study, data were retrospectively extracted from the gait and clinical database of a local hospital. Data of participants were included in the study if they were aged between 6 and 20 years at the time of treatment, received treatment according to the standard of care in the local hospital, and underwent CGA a year before and max 2 years after receiving their treatment Participants were excluded if they were not able to walk at least 10 m without a walking aid or orthosis (i.e. gross motor function classification system (GMFCS) level IV and higher), had further surgical intervention within the year, received botulinum toxin type A injection within 6 months prior to the pre-measurement, or if their clinical files were incomplete. The final dataset consisted of 119 participants with various neuromuscular and anatomical disorders, the vast majority (81 %) was diagnosed with CP (Table 1, Appendix A). Treatments consisted of orthotic management (ankle-foot orthosis, flexible or stiff), physiotherapy, botulinum toxin injections (botox), surgery, or any combination thereof. All these treatments had, according to the standard of care in the local hospital, the same goal: improving foot placement and heel-to-toe progression. The starting conditions were however not always comparable between participants. Therefore, the decision was made to not model each individual treatment but instead model the response to the hospitals’ standard of care. Participants were grouped based on their change in gait profile score (GPS, evaluated using mathematical formulations provided here: Tutorial on Gait Profile Score as control group age to calculate the GPS data from aged-matched typically developing children were used) from pre-to post-treatment CGA. If GPS increased by more than 1.6° participants were classified as “non-responders”, if the change was less than 1.6° they were classified as “responders” [17].

**Table 1:** **Participant overview.** Values are shown in mean ± SD (%). Diagnosis groups included 1. Spastic cerebral palsy, 2. Other types of CP, 3. Neuro-peripheral diseases such as spinal bifida or neuropathies, 4. Muscle disorders such as muscle dystrophy, 5. Skeletal deformities within foot or malalignment of the lower extremities, 6. Other such as genetical syndromes and idiopathic toe walking. Treatment groups included 1. Surgery: participants underwent a surgical intervention like Achilles tendon lengthening tibialis anterior shortening, multilevel surgery, bony foot surgery, 2. Botox: participants received botulin toxin type A injections 6 weeks in advance of the post-treatment clinical gait analysis within calf muscles or hamstrings, 3. Physiotherapy: participants received at least 2 h of physiotherapy per week between the pre- and post-treatment clinical gait analysis, 4. Orthosis: participants performed on the same day a clinical gait analysis barefoot and with orthosis (OSSA, AFO flex, AFO stiff) and shoes, all children were accustomed to walking with their orthosis (for further details on treatment, see appendix A). GMFCS: gross motor function classification system, GPS: gait profile score, non-responders: difference in GPS before and after treatment was −1.6°, stayed the same: difference in GPS before and after treatment was −1.6° < 1.6°, responders: difference in GPS before and after treatment was ≥+1.6°. Differences between groups were assessed using the student unpaired *t*-test (age, height, weight, GPS), chi-square test (diagnosis, GMFCS, treatment), or fisher exact test (sex) with α = 0.05 and * indicating significant differences were detected.

	Groups		
	Non-responders (Δ>1.6° GPS)	Responders (≤Δ1.6° GPS)	*p*
**Total**	28	91	
**Sex (m/f)**	18/10	61/27	0.65
**Age (years)**	11.9 ± 3.1	12.5 ± 3.8	0.42
**Height (m)**	1.45 ± 0.15	1.47 ± 0.18	0.61
**Weight (kg)**	39.9 ± 14.6	41.0 ± 16.1	0.76
**Diagnosis**			0.55
CP spastic	24	68	
CP other	1	5	
Neuro-peripheral	2	4	
Muscle	0	2	
Skeletal	1	3	
Other	0	9	
**Unilaterally involved**	16	40	
**Bilaterally involved**	12	51	
**GMFCS levels**			0.72
**I**	19	59	
**II**	9	30	
**III**	0	2	
**Treatment type**			0.42
Orthosis	12	28	
Physiotherapy	8	39	
Botox	4	8	
Surgery	4	16	
**GPS pre-therapy (°)**	10.7 ± 3.1	12.4 ± 5.0	0.096
**GPS post-therapy (°)**	13.0 ± 3.5	10.8 ± 3.3	0.004*

### 2.2 Data collection

Pre- and post-measurement, each participant underwent a physical examination, which included assessment of anthropometrics. Afterwards, reflective markers were placed on bony landmarks of the participant according to the conventional gait model 2.2 (CGM with 9.5 mm diameter markers, VICON, Oxford, UK [18]) and a 3D CGA was conducted. Here, subjects walked barefoot at their preferred walking speed on a 12 m instrumented walkway, equipped with an optoelectronic motion capture system (12-cameras MXT20, VICON, Oxford, UK, sampling rate 150Hz). Each participant performed at least 5 trials.

#### 2.2.1 Data processing

Pre-processing of the data (performed in VICON-NEXUS software package 2.8.2) included filtering of the data using the Woltring filter (CGM processing pipeline VICON-NEXUS, mean squared error set to 10 mm^2^ [19]). Further analyses were performed in MATLAB (MathWorks, Inc. Version R2019a, Natick, USA) with the open-source Biomechanical ToolKit package [20]. Trials with excessive soft tissue artefact, poor consistency, or signs of inaccurate marker placement were excluded. Gait events (initial contact and toe-off) were detected through automatic detection using the sagittal velocity approach [21]. The initial and the final captured stride and values that were more than 3 SD away from the mean for each individual were excluded as our interest lay with steady-state walking.

**Fig. 1:**
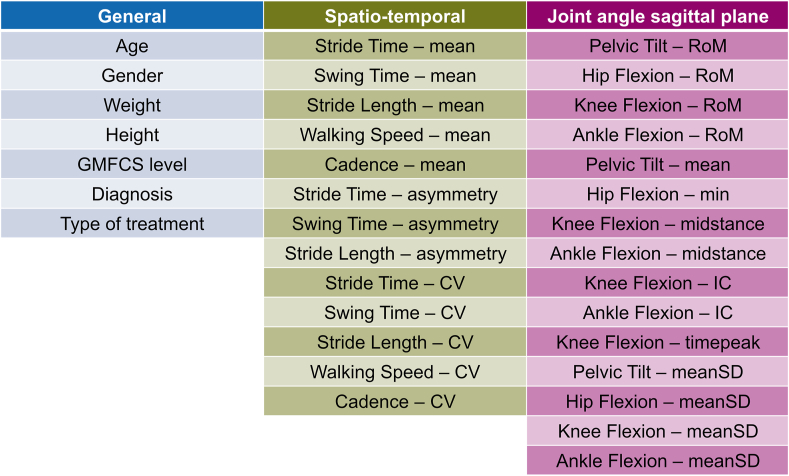
**Included features from pre-treatment clinical gait analysis.** Further information on features can be found in appendix B.

The extracted gait events were used to calculate spatio-temporal parameters (stride time – ST, stride length – SL, walking speed – WS, single support time – SS, cadence – CD), of which the variability was quantified through the coefficient of variation (CV). Gait events were also used to normalize the temporal axis of the kinematic curves from the CGM model to 100 % of the gait cycle. From the time normalized curves, the sagittal plane angles of the pelvis, hip, knee, and ankle were extracted, and their stride-to-stride fluctuations were quantified through meanSD [22], where the mean was taken from the variability (SD) between the trials at each time-point of the gait cycle. Discrete parameters were extracted (range of motion – RoM, average – mean, minimum – min, maximum – max, value at initial contact - IC and midstance, time of peak flexion - timepeak). For both CV and meanSD, the values were firstly computed separately for each limb and then combined to avoid the potentially confounding effect of limb asymmetry [23]. Asymmetry of gait was evaluated for SS (Asym-SS), ST (Asym-ST), and SL (Asym-SL) as a ratio between short and long for each stride [24]. In total, 35 features extracted from the pre-treatment CGA were included (Fig. 1, appendix B).

### 2.3 Analysis

Differences between the two groups “non-responders (increased GPS)” and “responders (same/reduced GPS)” were evaluated using Student's t-tests, chi-squared tests, or Fisher's exact tests depending on type of data, α was set at 0.05. Correlations between features and the change in GPS were evaluated using spearman correlations, only features with r < 0.6 were included. All features were normalized using z-scores, after which a principal component analysis (PCA) was applied to reduce the number of features, threshold was set at 80 % variance explained, leaving (Appendix C). These PCA components were used to train a support vector machine (SVM) with 5-fold cross-validation to detect participants who did not show any response in their GPS (<+1.6°) after receiving treatment. SVM was chosen as it is robust and ideal for classification-based problems, additionally it is effective in dealing with smaller sample sizes, and sufficiently uncomplicated to implement and tune. Bayesian optimization with 40 iterations was used to select hyper parameters, resulting in a linear SVM, with box constraint level 1, and 1 vs 1 approach. The procedure was performed within the MATLAB Classification Learner App (v2022a, MathWorks Inc., USA). The performance of the model was quantified by calculating accuracy, true positive rate (TPR), false negative rate (FNR), positive predictive value (PPV), and false discovery rate (FDR) for the group non-responsive to treatment. Additionally, feature importance scores were calculated using the chi-squared algorithm from the MATLAB Classification Learner App.

## 3 Results

28 out of 119 patients, increased their GPS after receiving standard treatment and were classified as “non-responders”, while 91 showed no change or a decreased GPS and were classified as “responders” (Table 1). No significant differences in sex, age, height, weight, diagnosis, treatment, or pre-treatment GPS were present between those who increased and those who maintained/reduced their GPS (t or fisher exact test p > 0.05). Those who were non-responders, increased their GPS from 10.7° ± 3.1–13.0° ± 3.5, while the others reduced their GPS from 12.4° ± 5.0–10.8° ± 3.3. Between features and change in GPS, no spearman correlations were above 0.6, therefore all features were included for the PCA analyses which resulted in 10 components for training the SVM.

**Fig. 2:**
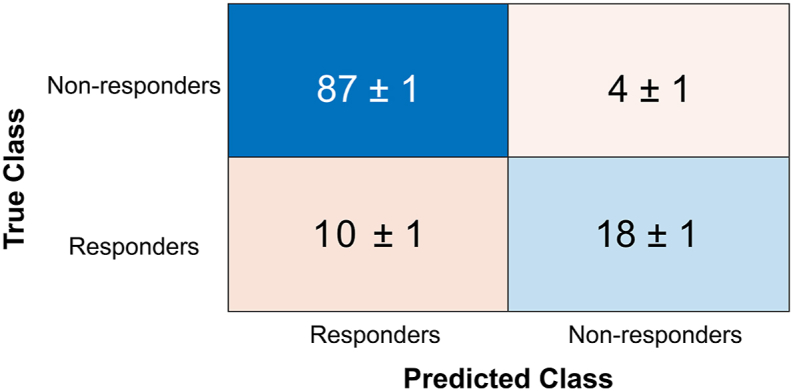
Average results for Confusion matrix from linear SVM, showing how many participants were classified correctly by the support vector machine on the diagonal.

An average accuracy of 88.2 ± 0.5 % was obtained, with AUC of 88.0 ± 1.0 % and 96.0 ± 0.9 % TPR for identifying those classified as at risk of non-responsive gait (Fig. 2, Table 2, Appendix D).

**Table 2:** Average results for linear SVM, reporting true positive rate (TPR), false negative rate (FNR), positive predictive value (PPV), and false discovery rate (FDR) in percentages.

	TPR	FNR	PPV	FDR
**Non-responders (%)**	96.0 ± 0.9	4.0 ± 0.9	83.2 ± 2.2	16.8 ± 2.2
**Responders (%)**	62.9 ± 4.3	37.1 ± 4.3	89.4 ± 1.0	10.6 ± 1.0

**Fig. 3:**
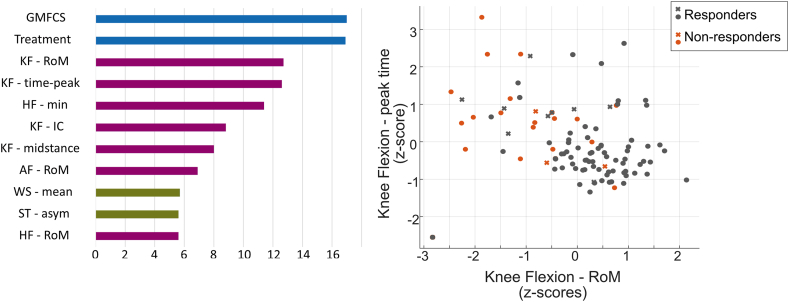
**Feature importance ranked through chi-square algorithm (A) and a scatter plot showing knee flexion range of motion (RoM) and time of peak knee flexion for each participant (B), with non-responders (increased GPS, orange), and responders (same/decreased GPS, grey) according to their change in GPS.** Those that are shown in round were correctly predicted by the model, whereas those once shown as a cross were incorrectly predicted. Only features with a relevance score of above 5 of the chi-squared feature relevance test are reported in the figure (for a complete list, see Appendix D). GPS: gait profile score.

The results of feature importance analysis using the built-in chi-squared algorithm, from the MATLAB Classification Learner App, showed that after GMFCS and treatment type, gait-based features on knee flexion – RoM, knee flexion – peak time, and hip flexion – minimum were the most influential features for the model (Fig. 3A).

## 4 Discussion

Paediatric movement disorders like cerebral palsy often negatively impact walking behaviour. Although clinical gait analysis is usually performed to guide treatment decisions, not all respond positively to their assigned treatment. Identifying these individuals based on their pre-treatment characteristics could alert clinicians and allow them to adapt towards a more personalized intervention. In this study, we investigated if a standard machine learning approach was able to identify children and adolescents suffering from movement disorders who might remain non-responsive in their gait characteristics after receiving standard treatment in Switzerland based on pre-treatment characteristics. In our study, treatment included orthotic management, physiotherapy, botulinum toxin injection, surgical intervention, or a combination of these approaches. A linear SVM was trained on routinely collected gait features and anthropometric data, yielding an average 96 % TPR for those who were non-responders with an AUC of 88 %. Feature importance analysis using the chi-squared algorithm revealed that GMFCS, treatment type, and range of motion of the knee were of the highest importance for the algorithm. If a participant has either extremely low range of motion for knee flexion or experiences later timing of peak knee flexion, the more likely they are to be classified as “at risk of non-responsive (or showing sub-optimal response) in their gait characteristics” after standard of care treatment (Fig. 3B).

This study included a multitude of treatments which makes it unique. Until now, research mainly focused on predicting the outcomes of specific interventions with accuracies reported between 56 and 78 % and sensitivities of up to 82 % [11,12,14]. However, such evaluations require large homogenous datasets, that are not frequently available in clinical settings. Therefore, this study used an alternative approach in which all different treatments were taken together as “the standard of care” as every individual received the care seen as most appropriate by the clinical staff at the time (period 2016–2020, one lead neuro-orthopaedic surgeon). This approach has allowed the development of the here presented “warning system”, that is able to identify individuals at risk of being classified as “non-responsive in their gait” irrespective of any one of the available standard treatments with 64 % true negative rate and 82 % negative prediction rate. No significant differences were seen between those who were classified as “increased” and those who were classified as “same/reduced” when looking at features individually (Table 1), but when taken together, the SVM was able to identify the assigned classes with an 88.2 % accuracy. Especially those individuals with relatively good GMFCS levels, but decreased knee flexion range of motion, and delayed peak knee flexion within the gait cycle showed a high risk of being classified as “non-responsive in their gait characteristics” when undergoing standard treatment. While problems with knee-flexion and the timing of peak knee flexion among children with cerebral palsy are well known [25], the current study highlights that these problems might in fact predispose individuals to further worsening of gait. In-depth future evaluations would be required to better understand why the GPS of these individuals did not respond positively or even worsened after the treatment, and what could be done to avoid this outcome.

Some limitations need to be acknowledged. Firstly, two features (asymmetry in stride and swing time) showed low significant correlation (with r = −0.20 and −0.17 respectively) with the true label, the change in GPS. As these correlations were low, we deemed these unlikely to interfere with the model's performance. What should be acknowledged, is that only linear correlations, this was because a linear SVM was applied, making it unlikely that other types of correlations would have influenced the model. Secondly there might be bias associated with the true label, it might not always reflect the objective true value. It could, for example, be that an individual would have deteriorated even further if he or she had not received treatment, making a small deterioration of GPS not necessarily a bad outcome. It was unfortunately not possible to accurately estimate what the “natural” progression would have been without treatment. Therefore, GPS was considered the optimal choice, as it is one of the scores where the minimal clinically relevant change has previously been established [17] – allowing objective categorization. It is however, important to note that a GPS of 1.6° is rather small value given the volume covered during data-collection and the labels should therefore be interpreted with caution. In the future, it would be interesting to compare the change in GPS with patient-reported outcome measures. Third, the amount of CGA features included in the current model were limited. During CGA, a high variety of data is collected. Next to kinematic and anthropometric features, kinetic and electromyography features could also be considered to extend the presented model and possibly further improve its performance as well as interpretability. As not all clinical gait laboratories have access to force plates and electromyographic systems, the current study focussed on widely used kinematic and anthropometric features that have shown promising results in previous investigations [[11], [12], [13], [14], [15], [16],^26^]. Last but not least, current CGA focusses only on the mechanical part of gait while gait patterns and reactions to treatment also depend on neuromotor control and cognitive abilities as well as external factors that are currently not considered by our model. This may explain at least some part of the inaccuracies of our results. In this respect, we may have to accept a certain imprecision in predicting the outcome as it will be difficult even in the future to achieve data that can comprehensively cover all relevant domains in children with cerebral palsy.

Despite these limitations, we can conclude that via the use of classical machine learning approaches, our study was able to identify patient specific features that predispose them to a risk of gait characteristics not responding after treatment, based solely on routinely collected gait features and anthropometrics. While further evaluations are certainly required before such models can be deployed clinical setting, this study serves as a first step towards implementing a warning system that could potentially inform and notify clinicians if a certain patient might be at risk of poor functional outcomes and would require more attention.

## Data availability statement

The normalized datasets are available in the supplementary material, see appendix D. For further details on the dataset, the corresponding author can be contacted.

## Ethics statement

This retrospective study involving human participants was reviewed and approved by the Cantonal Ethics Committee, Zurich, Switzerland (KEK 2018-01640). Written general informed consent to allow usage of the collected data for retrospective studies was provided by each participant and/or each participant's legal guardian/next of kin.

## Funding

This study was financially supported by the Ralf-LoddenkemperFoundation under grant CH-270.7.002.704-3.

## CRediT authorship contribution statement

**Rosa M.S. Visscher:** Conceptualization, Data curation, Formal analysis, Investigation, Methodology, Project administration, Supervision, Visualization, Writing – original draft, Writing – review & editing. **Julia Murer:** Formal analysis, Investigation, Writing – review & editing. **Fatemeh Fahimi:** Investigation, Methodology, Writing – original draft, Writing – review & editing. **Elke Viehweger:** Project administration, Supervision, Writing – review & editing. **William R. Taylor:** Project administration, Supervision, Writing – review & editing. **Reinald Brunner:** Conceptualization, Project administration, Supervision, Writing – review & editing. **Navrag Singh:** Conceptualization, Methodology, Supervision, Writing – original draft, Writing – review & editing.

## Declaration of competing interest

None Declared.
