# Supplementary material for: Identifying treatment non-responders based on pre-treatment gait characteristics - A machine learning approach
Source: Heliyon. 2023 Oct 23;9(11):e21242. doi: 10.1016/j.heliyon.2023.e21242 (PMC10613900; doi:10.1016/j.heliyon.2023.e21242)
Supplement: Multimedia component 2 [file mmc2.docx]

**Appendix B: Data and Feature Specification**

For the clinical data, some features were summarized into different classes to be usable as a

feature for the classifier. All kinematic parameters (green) and joint angles (orange) the

following way: First the mean of a certain feature for each leg was calculated and then the

mean of the two legs was derived.

To calculate the asymmetry of the gait parameters (GA), first it was determined which foot

had a longer and which a shorter step (time/length): 1

$$Asymmetry= \left| \frac{short}{long} \right| \times100\%$$

The coefficient of variation (CV) was calculated by dividing the standard deviation by the mean

and is multiplied with 100 to receive a percentage (%):

$$CV= \left( \frac{SD}{\bar{x}} \right) \times100\%$$

The mean SD was calculated using the following formula:

$$MeanSD \left( \Delta x \right)= \left\langle{SD}_{i}\left[ x \right] \right\rangle for i \in\left\{ 0, \ldots, 100\% \right\}$$

| **Feature name** | **Definition** |
| --- | --- |
| Treatment type | Summarized in four categories  - 1: Orthosis  - 2: Physio  - 3: Surgical procedures  - 4: Botox (BTX-A) |
| Age | In years measured at pre-measurement |
| Gender | Two classes:  - 1: male  - 2: female |
| Weight | Measured in kg at pre-measurement |
| Height | Measured in m at pre-measurement |
| GMFCS | Only level I to level III were included:  - 1: GMFCS level 1  - 2: GMFCS level 2  - 3: GMFCS level 3 |
| Diagnosis | Summarized in eight categories  - 1: Spastic CP  - 2: non-spastic CP  - 3: Spinal Cord Injury (SCI), Spinal  bifida, Neuropathy  - 4: Muscle diseases, Muscle  dystrophy  - 5: Traumatic Brain Injury (TBI),  Microcephaly  - 6: Ideopathic Toe walking (ITW),  Other  - 7: Various genetic syndromes that  cause movement disorders  - 8: Skeletal knee, Skeletal Foot,  Skeletal malalignment |
| Stride Time – mean | Time between IC of one foot and the next IC  of the same foot averaged across all  strides |
| Swing Time – mean | Time during the foot is not in contact with  the ground  à time between TO and next IC of the  same foot averaged across all strides |
| Stride Length – mean | Distance between IC of one foot and the  next IC of the same foot averaged across  all strides |
| Cadence – mean | Total number of gait cycles in a given time  period |
| Walking Speed – mean | Distance covered by the whole body in a  given time |
| Stride Time – asymmetry | Ratio between shorter and longer swing  time for each consecutive pair of strides |
| Swing Time – asymmetry | Ratio between shorter and longer stride  time for each consecutive pair of strides |
| Stride Length – asymmetry | Ratio between shorter and longer stride  length for each consecutive pair of strides |
| Stride Time – cv | Coefficient of variation of time between two consecutive ICs on  the same side |
| Swing Time – cv | Coefficient of variation of time when only one foot was in  contact with the ground |
| Stride Length – cv | Coefficient of variation of distance travelled between two  consecutive ICs of the same side |
| Cadence – cv | Coefficient of variation of number of strides in a given time |
| Walking Speed – cv | Coefficient of variation of distance covered by the whole body  in a given time |
| Pelvic tilt – rom | Range of motion of pelvis in sagittal plane over course of gait cycle |
| Hip flexion – rom | Range of motion of hip in sagittal plane over course of gait cycle |
| Knee flexion – rom | Range of motion of knee in sagittal plane over course of gait cycle |
| Ankle flexion – rom | Range of motion of ankle in sagittal plane over course of gait cycle |
| Pelvic tilt – mean | Average pelvic tilt over course of gait cycle |
| Hip flexion – min | Minimal amount of hip flexion over course of gait cycle |
| Knee flexion – ic | Amount of knee flexion at initial contact |
| Ankle flexion – ic | Amount of ankle-dorsiflexion at initial contact |
| Knee flexion – midstance | Amount of knee flexion at midstance |
| Ankle flexion – midstance | Amount of ankle-dorsiflexion at midstance |
| Pelvic tilt – meansd | MeanSD of the rotation of the pelvic tilt in  the sagittal plane, normalized to the gait  cycle |
| Hip flexion – meansd | MeanSD of the rotation of the hip in the  sagittal plane, normalized to the gait cycle |
| Knee flexion – meansd | MeanSD of the rotation of the knee in the  sagittal plane, normalized to the gait cycle |
| Ankle flexion – meansd | MeanSD of the rotation of the ankle in the  sagittal plane, normalized to the gait cycle |
