# Supplementary material for: Identifying treatment non-responders based on pre-treatment gait characteristics - A machine learning approach
Source: Heliyon. 2023 Oct 23;9(11):e21242. doi: 10.1016/j.heliyon.2023.e21242 (PMC10613900; doi:10.1016/j.heliyon.2023.e21242)
Supplement: Multimedia component 3 [file mmc3.docx]

**Appendix C: Principal component analysis**

To reduce the input features, a standard principal component analysis was conducted using MATLAB (v2022a, MathWorks Inc., USA). Threshold was placed at 80% variance explained; therefore, the first 10 components were included for training the model (Figure 1).


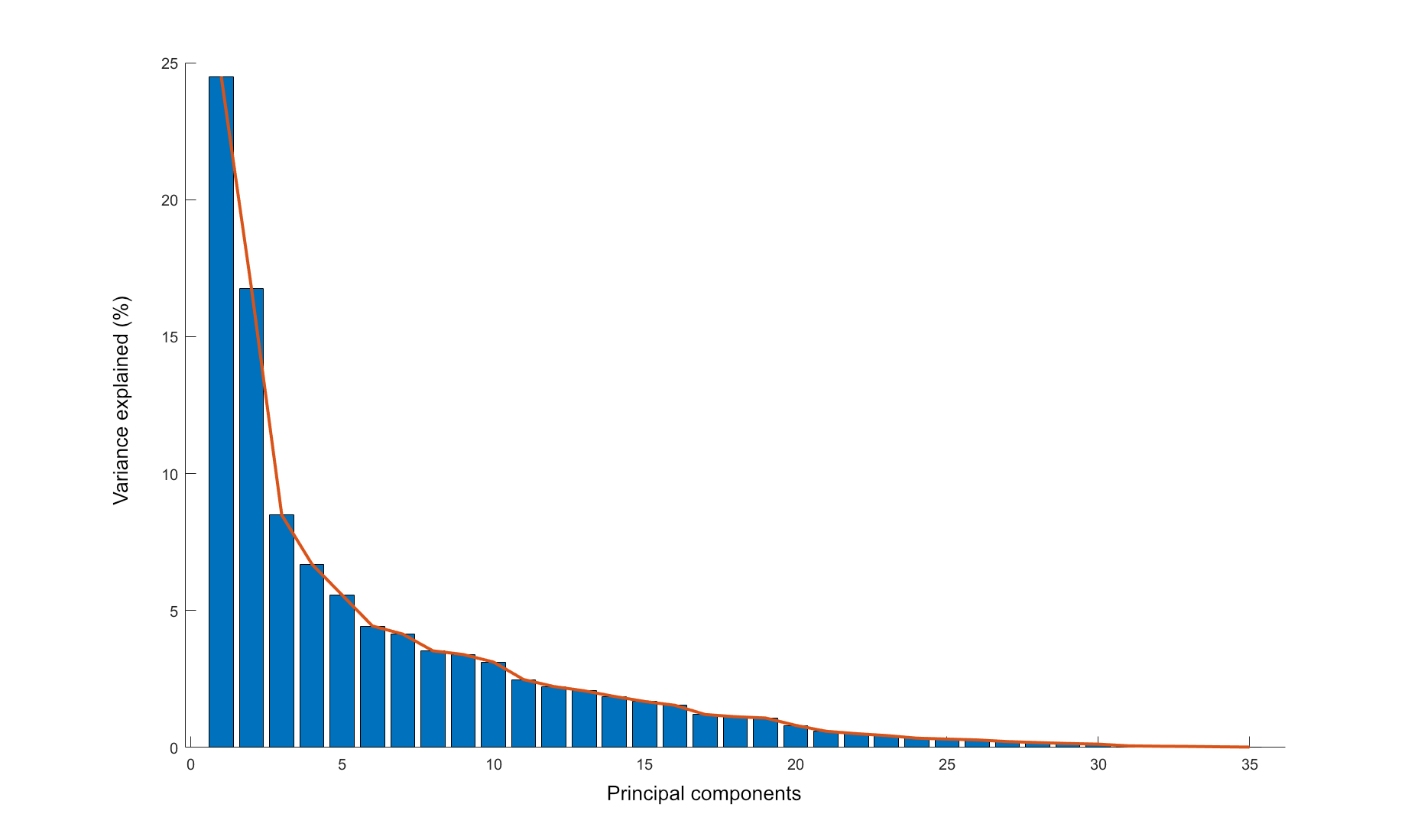


Figure 1: total variance explained by principal components
